# Wheat aroma biomarkers induced by saline-alkali soil: based on HS-SPME-GC-MS and molecular docking

**DOI:** 10.3389/fnut.2026.1788319

**Published:** 2026-03-04

**Authors:** Jiarong Zhang, Hao Wang, Zihe Wang, Bo Zhang, Huijing Li, Qianqian Sun

**Affiliations:** 1 College of Food Science and Technology, Hebei Agricultural University, Baoding, China; 2 Institute of Agricultural Product Processing and Nutritional Health, Chinese Academy of Agricultural Sciences (Cangzhou)/Hebei Key Laboratory of Drought-Alkali Tolerance in Wheat, Cangzhou, China; 3 Zhongyuan Research Center, Chinese Academy of Agricultural Sciences, Xinxiang, China; 4 Institute of Food Science and Technology, Chinese Academy of Agricultural Sciences/Comprehensive Utilization Laboratory of Cereal and Oil Processing, Ministry of Agriculture and Rural, Beijing, China

**Keywords:** gas chromatography-mass spectrometry (GC-MS), molecular docking, relative odor activity values (ROAVs), saline-alkali (SA) soil, volatile organic compounds (VOCs), wheat aroma

## Abstract

**Background:**

The precise volatile compounds accountable for the characteristic scent of the wheat aroma were presently unidentified. Given the prominent manifestation of “wheat aroma” in wheat cultivated in saline-alkali (SA) soil, five wheat varieties planted in saline-alkali soil and control soil (low saline-alkali soil) to create “rich-aroma” and “bland-aroma” samples, respectively.

**Methods:**

The volatile profiles of these two groups of samples were analyzed using headspace solid-phase microextraction coupled with gas chromatographyed with gas chromathes-SPME-GC-MS), and differential characteristic volatile compounds between “rich-aroma” and “bland-aroma” samples were identified by combining paired *t*-tests, relative odor activity values (ROAVs), and molecular docking analysis.

**Results:**

A total of 75 volatile compounds were identified using HS-SPME-GC-MS. Paired *t*-test result revealed that a significant increase (*p* < 0.05) in the peak areas and relative contents of limonene (>109.74% increase), β-pinene (> 474.09% increase) contrasted with reduced butanoic acid (>38.71% reduction) and (E,E)-3,5-octadien-2-one (>16.05% reduction) in saline-alkali soil-cultivated wheats. Molecular docking demonstrated high binding energies (<-4.25 kcal/mol) of limonene, β-pinene, and (E,E)-3,5-octadien-2-one to olfactory receptors, corroborated by relative odor activity values (ROAVs. > 1).

**Conclusion:**

Limonene, β-pinene and (E,E)-3,5-octadien-2-one are identified as possible saline-alkali soil-induced wheat aroma biomarkers, and offer theoretical insights for verificating wheat aroma. Identifying potential characteristic biomarkers in wheat under saline-alkali stress provides important theoretical insights for improving the flavor characteristics of wheat.

## 1 Introduction

Saline-alkali soil leads to soil compaction and nutrient imbalance, hampering wheat growth (1). However, fully exploiting the utilization pathways of saline-alkali soil can not only provide complementary grain production but also yield distinctive crops, thereby enhancing agricultural biodiversity and economic value while contributing to food security. China possesses substantial areas of utilizable saline-alkali soil. How to utilize these lands has become a focus of attention. Notably, sensory evaluations by Cangzhou residents suggest that wheat grown in SA soil exhibits a distinct and intense “wheat aroma” (2), thereby enhancing its market appeal and added value (3, 4). However, the specific chemical alterations responsible for the enhancement of its wheat aroma remain unclear.

The wheat aroma is composed of volatile compounds such as aldehydes, alcohols, esters, ketones and terpenes, and its formation involves complex biosynthesis. The formation of wheat aroma involves complex biosynthetic pathways. During growth, ripening and post-ripening stages of wheat grains, enzyme-mediated carbohydrate, fatty acid and protein metabolism generate diverse volatile organic compounds (VOCs), which collectively define wheat aroma profiles (5). Studies on wheat flour have consistently identified lipid-derived aldehydes such as hexanal, nonanal and (E)-2-non-enal, together with cereal-like volatiles including 2-pentylfuran and 1-octen-3-ol, as key contributors to green, fatty and grain-like notes in wheat grain and flour (6). In addition, benzaldehyde and 2-acetyl-1-pyrroline, although present at lower concentrations, exhibit high odor potency and contribute almond-like, roasted and popcorn-like nuances (7).

The biosynthesis of these VOCs is profoundly influenced by abiotic stress conditions. Such stresses alter the pools of volatile precursors such as amino acids (e.g., glutamic acid and proline) (8), unsaturated fatty acids (e.g., linoleic acid) (9), and soluble sugars (e.g., glucose, fructose and sucrose) (10) by triggering osmotic adjustment and oxidative stress responses. Concurrently, signaling pathways based on reactive oxygen species (11) and phytohormones (12) regulate the expression of key enzyme genes, thereby selectively promoting the formation of specific aldehydes, lactones, ketones and terpenoids like limonene (13), β-ocimene (14), and β-pinene (15). This metabolic reprograming fundamentally reshapes the aroma profile of the plant. For instance, mild drought stress has been shown to significantly increase the diversity of VOCs in tea leaves, elevating the concentrations of low-threshold VOCs associated with sweet, floral, and fruity aromas (16). Similarly, salt stress (e.g., seawater irrigation in grapes) enhances the accumulation of volatiles derived from fatty acid and isoprene pathways, including terpenes such as citronellol and myrcene (17). While the influence of abiotic stress on cereal aroma has predominantly focused on rice—where moderate salt stress enhances the characteristic aroma by modulating the biosynthesis of 2-acetyl-1-pyrroline through altered amino acid metabolism and lipid peroxidation (18)—far less attention has been paid to wheat. Our previous research found that the characteristics of wheat under saline-alkali stress are elevated 1-butanol and 1-octen-3-ol but reduced ethyl 3-methylbutyrate and 1-octen-3-one by gas chromatography-ionmobility spectrometry (GC-IMS) (19). However, GC-IMS exhibits a certain selectivity or limited detection range for compounds, meaning that the aforementioned four compounds may represent only a fraction of the complex aroma profile. To capture the complete VOC profile more comprehensively and without biasy modulating the biosynthesis of 2ehydes, lac Dsity and economic value while contrib-IMSpture the complete VOC profilgas chromatography-mass spectrometry (GC-MS) for sample analysis. GC-MS offers broader compound identification capabilities, higher sensitivity, and robust spectral library support (20, 21), facilitating the discovery of more potential aroma-active compounds. This study employs an intensity-graded sampling strategy, classifying five wheat varieties grown in both SA and control soils into “rich-aroma” and “bland-aroma” groups. Therefore, the varieties are repetitive, this study does not focus on the differences brought by different genotypes. Using paired HS-SPME-GC-MS profiling, molecular docking, and ROAV analysis, this study aims to (1) Identify differential VOCs in SA-stressed wheat and select potential signature biomarkers; and (2) elucidate their molecular interactions with olfactory receptors. This integrated approach advances understanding of abiotic stress-induced aroma formation, and provides essential theoretical insights for improving wheat flavor profiles.

## 2 Materials and methods

### 2.1 Sample collection

In the year 2022, five distinct wheat cultivars—Cangmai 6002 (C6002), Cangmai 6005 (C6005), Jimai 22 (JM22), Shiluan 02-1 (SL02-1), and Malannuomai NO.1 (N01)—were collected from two locations in Cangzhou, Hebei Province, China, namely Xi Huayuan (XHY) and Zhong Jie village (ZJ), which are 50 km apart in a straight line, with basically the same climate conditions but different longitude and latitude and soil salinity and alkalinity (Table 1). Among these cultivars, C6002 and C6005 are medium gluten wheat varieties with moderate tolerance to salinity-alkalinity, whereas JM22, SL02-1 and N01 are categorized as medium-gluten, strong-gluten, and waxy wheat varieties, respectively, all of which are sensitive to salinity-alkalinity stress, and are the main cultivated varieties in the local area (Supplementary Table 1). The five wheat varieties were assigned randomly to plots in each location following a randomized complete block design, with each plot covering an area of 666.67 m^2^ (1 *mu*).

**TABLE 1:** The pH and total salt content of soil in wheat cultivation sites.

The cultivation site	longitude and latitude	Soil horizon [cm]	pH (mean ± s.d.)	Total salt content (mean ± s.d.)[g/kg]	Abbreviation
Xi Huayuan	E:116°52′38.22″ N:38°22′42.38″	0-40	7.76 ± 0.07	1.23 ± 0.03	XHY
Zhong Jie	E:117°28′16.99″ N:38°24′1.75″	0-40	8.34 ± 0.20**	2.54 ± 0.35**	ZJ

The pH and total salt content for the sampling sites was provided by the Academy of Agriculture and Forestry Sciences, Hebei Key Laboratory of Drought–Alkali Tolerance in Wheat, Cangzhou, China.“**” represents a significant difference (*p* < 0.01).

After the wheat had fully matured, they were collected using an Austrian Wintersteiger classic combine harvester. From each sample, approximately 5 kilograms were randomly selected and labeled. Then, three separate 100-gram portions were drawn at random from each 5-kilogram sample. A total of 30 samples were obtained (2 fields × 5 genotypes × 3 replicates), and all were processed using a versatile grinder (model ZK-100B, manufactured by Zhongke Haoyu Technology Co., Ltd., China). After processing, the samples were placed in sealed bags and kept at -20°C for subsequent use.

### 2.2 Gas chromatography-mass spectrometry analysis

The extraction and analysis of VOCs was conducted according to the published experimental scheme, with some improvements (22). The headspace solid-phase microextraction (HS-SPME) technique was employed to extract VOCs. Wheat wholemeal samples (2 g) were placed in 20 mL vials, equilibrated at 60°C for 40 min, and extracted with HS-SPME for 80 min at 60°C. The extracted fiber was 50/30 μm divinylbenzene/carboxenTM/polydimethylsiloxane (DVB/CAR/PDMS) from Supelco (Bellefonte, PA, United States).

Analysis of the VOCs was performed using GC-MS (TRACE1300, ISQ9000, United States), employing a DB-Wax capillary column (30 m × 0.25 mm × 0.25 μm). Post-extraction, the fiber was desorbed into the GC injector port for 2 min at 250°C in splitless mode. Helium at a flow rate of 1 mL/min was used as the carrier gas. The oven temperature was initially set at 40°C for 5 min, then ramped at 5°C/min to 220°C for 10 min. The MS was operated in electron ionization mode (EI) at 70 eV, with a multiplier voltage of 1247 V. The ion source temperature was maintained at 200°C, and the interface temperature at 250°C. Full scan mode was employed to detect VOCs across a mass range from m/z 35 to 600. Each sample underwent triplicate analysis.

### 2.3 Identification of volatile organic compounds

Compounds identification relied on comparison of their mass spectra with those in the NIST 20 library. VOCs with Match Factor greater than 750. Additionally, retention index was calculated using Kovats retention index based on a series of straight-chain alkanes (C7-C27) under the aforementioned chromatographic conditions.

To confirm GC-MS identifications (Table 2), the standards (reference compounds) of 2-pentyl-furan (3777-69-3, ≥ 98%), 1-hexanol (111-27-3, ≥ 99%), 1-octen-3-ol (3391-86-4, ≥ 98.04%) and limonene (138-86-3, 1,000 μg/mL) were selected. These standards were diluted and mixed with high-quality chromatographic grade methanol (99.9%). A stock solution containing a concentration of 50 ppm for the mixed standards was prepared. The sources of 2-pentyl-furan, 1-hexanol, 1-octen-3-ol and methanol were Shanghai Macklin Biochemical Co., Ltd., China, while the source of Limonene was Tan-Mo Technology Co., Ltd., China.

**TABLE 2:** Volatile organic compounds identified by GC-MS.

No.	Classification	Volatile organic compounds	Molecular formula	CAS No.	Retention index (Exp)	Retention index (Lit)
1	Alcohols(30.05%∼36.13%)	1-Propanol, 2-methyl-	C_4_H_10_O	78-83-1	1,091	-
2		1-Butanol, 3-methyl-	C_5_H_12_O	70907-83-4	1,213	1,209
3		1-Pentanol	C_5_H_12_O	71-41-0	1,248	1,250
4		1-Hexanol	C_6_H_14_O	111-27-3	1,353	1,355
5		2-Hexen-1-ol, (Z)-	C_6_H_12_O	928-94-9	1,403	1,407
6		1-Octen-3-ol	C_8_H_16_O	3391-86-4	1,450	1,450
7		1-Heptanol	C_7_H_16_O	111-70-6	1,453	1,453
8		Linalyl oxide	C_10_H_18_O_2_	60047-17-8	1,466	1,467
9		1-Hexanol, 2-ethyl-	C_8_H_18_O	104-76-7	1,495	1,491
10		2,3-Butanediol	C_4_H_10_O_2_	513-85-9	1,546	1,549
11		1-Octanol	C_8_H_18_O	111-87-5	1,557	1,559
12		Terpinen-4-ol	C_10_H_18_O	562-74-3	1,606	1,602
13		Benzyl alcohol	C_7_H_8_O	100-51-6	1,882	1,887
1	Alkanes (22.59%∼26.55%)	Pentane, 3,3-dimethyl-	C_7_H_16_	562-49-2	1,039	-
2		Hexane,2,2,5,5-tetramethyl-	C_10_H_22_	1071-81-4	1,159	-
3		Heptane, 3-ethyl-5-methyl-	C_10_H_22_	52896-90-9	1,242	-
4		Dodecane, 4-methyl-	C_13_H_28_	6117-97-1	1,256	-
5		Octane, 2,3,6-trimethyl-	C_11_H_24_	62016-33-5	1,279	-
6		Tridecane	C_13_H_28_	629-50-5	1,300	1,300
7		Dodecane, 4,6-dimethyl-	C_14_H_30_	61141-72-8	1,358	-
8		Tetradecane	C_14_H_30_	629-59-4	1,400	1,400
9		Pentadecane	C_15_H_32_	629-62-9	1,500	1,500
10		Heptadecane	C_17_H_36_	629-78-7	1,700	-
1	Terpenes (0.81∼16.41%)	β-pinene	C_10_H_16_	692-96-6	1,107	1,108
2		Limonene	C_10_H_16_	127-91-3	1,200	1,199
1	Aromatic compounds(9.39%∼15.73%)	Benzene, 1,3-dimethyl-	C_8_H_10_	108-38-3	1,145	1,143
2		o-Xylene	C_8_H_10_	95-47-6	1,188	1,187
3		Styrene	C_8_H_8_	100-42-5	1,263	1,261
4		p-Cymene	C_10_H_14_	99-87-6	1,268	-
5		Benzene,1-methyl-4-propyl-	C_10_H_14_	1074-17-5	1,310	1,312
6		Naphthalene	C_10_H_8_	91-20-3	1,751	1,745
7		Benzene,1-(1,5-dimethyl-4-hexenyl)-4-methyl-	C_15_H_22_	644-30-4	1,778	1,777
8		Naphthalene, 2-methyl-	C_11_H_10_	91-57-6	1,863	1,856
9		Naphthalene, 1-methyl-	C_11_H_10_	90-12-0	1,898	1,884
10		1,8-dimethyl-Naphthalene	C_12_H_12_	569-41-5	1,973	1,989
11		1,6-dimethyl-Naphthalene	C_12_H_12_	575-43-9	2,003	2,006
12		2-Methoxy-4-vinylphenol	C_9_H_10_O_2_	7786-61-0	2,205	2,220
13		2,4-Di-tert-butylphenol	C_14_H_22_O	96-76-4	2,317	2,330
1	Acids (5.56∼9.08%)	Acetic acid	C_2_H_4_O_2_	64-19-7	1,444	1,449
2		Butanoic acid	C_4_H_8_O_2_	107-92-6	1,627	-
3		Butanoic acid, 3-methyl-	C_5_H_10_O_2_	503-74-2	1,659	1,662
4		Hexanoic acid	C_6_H_12_O_2_	142-62-1	1,851	1,846
5		Heptanoic acid	C_7_H_14_O_2_	111-14-8	1,957	1,950
6		Octanoic acid	C_8_H_16_O_2_	124-07-2	2,064	2,060
7		Non-anoic acid	C_9_H_18_O_2_	112-05-0	2,170	2,170
8		Undecanoic acid, 2-methyl-	C_12_H_24_O_2_	24323-25-9	2,218	-
1	Esters (3.11∼3.79%)	Octanoic acid, methyl ester	C_9_H_18_O_2_	111-11-5	1,394	-
2		Non-anoic acid, methyl ester	C_10_H_20_O_2_	1731-84-6	1,503	1,499
3		gamma-Valerolactone	C_10_H_18_O_2_	2890-67-7	1,613	-
4		Butyrolactone	C_4_H_6_O_2_	96-48-0	1,638	-
5		Lavender lactone	C_7_H_10_O_2_	1073-11-6	1,680	1,682
6		gamma-Hexalactone	C_6_H_10_O_2_	695-06-7	1,688	1,694
7		Dodecanoic acid, methyl ester	C_13_H_26_O_2_	111-82-0	1,802	1,804
8		gamma-Non-anolactone	C_9_H_16_O_2_	104-61-0	2,040	2,040
1	Aldehydes (2.26∼4.63%)	Non-anal	C_9_H_18_O	124-19-6	1,398	1,401
2		2,4-Hexadienal	C_6_H_8_O	142-83-6	1,409	1,410
3		2-Octenal, (E)-	C_8_H_14_O	2548-87-0	1,435	1,439
4		2-Non-enal, (E)-	C_9_H_16_O	18829-56-6	1,541	1,545
5		2,6-Non-adienal	C_9_H_14_O	557-48-2	1,598	1,599
6		2-Decenal, (E)-	C_10_H_18_O	3913-81-3	1,649	1,653
7		2,4-Non-adienal, (E,E)-	C_9_H_14_O	5910-87-2	1,708	1,714
8		2-Undecenal	C_11_H_20_O	2463-77-6	1,757	1,755
9		2,4-Decadienal, (E,E)-	C_10_H_16_O	25152-84-5	1,817	1,823
10		Vanillin	C_8_H_8_O_3_	121-33-5	2,585	2,580
1	Ketones(1.75∼2.79%)	2-Octanone	C_8_H_16_O	111-13-7	1,289	1,287
2		5-Hepten-2-one, 6-methyl-	C_8_H_14_O	110-93-0	1,342	1,345
3		3-Octen-2-one	C_8_H_14_O	1669-44-9	1,412	1,411
4		5-Octen-4-one, 7-methyl-	C_9_H_16_O	32064-78-1	1,469	–
5		(E,E)-3,5-Octadien-2-one	C_8_H_12_O	30086-02-3	1,576	1,570
6		2-Undecanone	C_11_H_22_O	112-12-9	1,602	1,598
7		5,9-Undecadien-2-one, 6,10- dimethyl-, (E)-	C_13_H_22_O	3796-70-1	1,859	1,859
1	Olefins	1-Methylcycloheptene	C_8_H_14_	55308-20-8	1,703	-
2	(0.12%∼0.26%)	3-Heptene, 2- methyl-, (E)-	C_8_H_16_	692-96-6	1,761	-
1	Other compounds (3.66∼9.47%)	Furan, 2-pentyl-	C_9_H_14_O	3777-69-3	1,236	1,239
2		Benzothiazole	C_7_H_5_NS	95-16-9	1,965	1,958

Retention Index (Exp) indicates experimental linear retention index; Retention Index (Lit) indicates retention index reported for reported in the literature. “-” indicates that no appropriate reference value was retrieved under similar chromatographic conditions.

An aliquot of 1 mL from the above-prepared mixed standard sample stock solution was extracted into a headspace bottle with a volume of 20 mL. Then it was analyzed by HS-SPME-GC-MS using the same conditions as the previous tests. The reliability of VOCs identification results in this study can be considered satisfactory, if the retention times for the aforementioned reference compounds in both standard and test samples were essentially identical. The confirmation results are presented in Supplementary Figure 1.

### 2.4 Molecular docking

The method was adapted from to Jia et al. (23) with minor modifications. This study investigated interactions between sixteen significant human olfactory receptors (OR51E1, OR52D1, OR1D2, OR7C1, OR8B3, OR6F1, OR13C3, OR10A6, OR5P3, OR3A1, OR14A2, OR9G1, OR11H6, OR2W1, OR11H4, and OR4Q3) and impotent VOCs. Protein structures for the olfactory receptors were sourced from UniProt and AlphaFold. SDF files for each small molecule ligand were obtained from the PubChem database and converted to PDB format using PyMOL 2.3.0. Molecular docking was performed using AutoDockTools (ADT) v1.5.7 and AutoDock Vina v1.2.0, prioritizing receptor-ligand complexes prioritized based on low binding energy rankings. The results were further analyzed using PyMOL and LigPlot for interaction visualization.

### 2.5 Relative odor activity values of volatile compounds analysis

The ROAV was a very useful tool for assessing the contribution of each aroma compound to the overall flavor profile (24). The larger the ROAV, the greater the contribution to the overall flavor of the sample.

The ROAVs. of each component were calculated to analyze its contribution of each component to the overall wheat aroma. The formula was as follows:

*ROAV* = 100 × *C%_*A*_*/C%_*max*_ × T_*max*_/*T*_*A*_

Where, *C%_*A*_* and *T*_*A*_ were the relative percentage content and odor threshold concentration (Table 3) of the compound, respectively. C%_*max*_ and T_*max*_ were the relative percentage content and odor threshold concentration (Table 3) of the component that contributes the most to the overall flavor of the sample, respectively.

**TABLE 3:** ROAVs calculation of key volatile organic compounds of wheat cultivated in XHY and ZJ.

Volatile organic compounds	Sensory threshold(μ g/kg)	C6002		C6005		JM22		N01		SL02-1		Odor description
		XHY	ZJ	XHY	ZJ	XHY	ZJ	XHY	ZJ	XHY	ZJ	
Limonene	200	100.00	100.00	100.00	100.00	21.20	100.00	100.00	100.00	100.00	100.00	Sweet, orange
β-pinene	140	7.21	23.28	1.61	15.99	29.31	35.77	-	2.37	0.54	5.56	Pine, resin, turpentine
(E,E)-3,5-octadien-2-one	100	14.99	9.99	24.80	10.67	100.00	21.45	47.11	14.02	33.98	10.53	Fatty

“ - ” indicates that the system is not detected.

### 2.6 Data analysis

All experiments were conducted in triplicates and the data are presented as the mean ± standard deviation. The data treatment was carried out using the SPSS 23.0 package software (SPSS Inc., Chicago, IL, United States). *T*-tests were performed to analyze significant differences in VOCs of wheat varieties cultivated in various cultivation sites. The bar graph and heat map were created using Origin 2024 software (OriginLab Corporation, Northampton, MA). Principal component analysis (PCA) and linear discriminant analysis (LDA) were utilized for data pattern identification and analysis.

## 3 Results

### 3.1 Volatile organic compounds analysis by gas chromatography-mass spectrometry

The composition and contents of VOCs in five wheat varieties cultivated in SA and control soils were compared by GC-MS. A total of 75 VOCs were identified and classified into 11 chemical classes namely: alcohols, hydrocarbons, terpenes, aromatic compounds, acids, ethers, esters, aldehydes, ketones and heterocyclic compounds. Calculate the proportion of the peak area of each type of substance in all substances in each sample (Table 2). Among these classes, alcohols, alkanes, terpenes, aromatic compounds, acids, esters, aldehydes and the remaining substances accounted for 30.05–36.13%, 22.59–26.55%, 0.81–16.41%, 9.39–15.73%, 5.56–9.08%, 3.11–3.79%, 2.26–4.63%, and 5.53–12.52% of the total VOCs in the wheat varieties, respectively.

**FIGURE 1:**
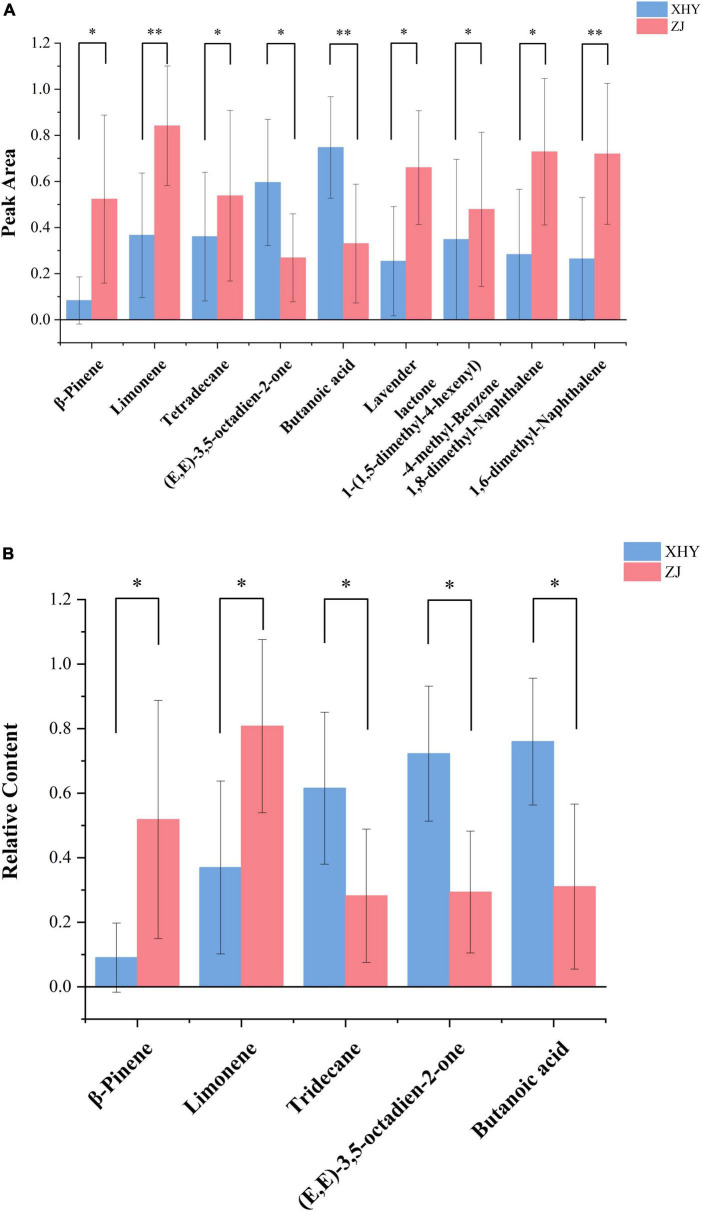
Bar chart of compounds with significantly different peak areas **(A)** and relative contents **(B)** in wheat cultivated at two sites (Xi Huayuan and Zhong Jie). * and ** represent the significant difference (*p* < 0.05) and (*p* < 0.01) respectively. All data were normalized using Min-Max.

Paired *t*-tests demonstrated significant alterations in the volatile profiles of saline-alkali (SA) soil-cultivated wheat versus control samples (Figure 1A). Under SA cultivation, seven compounds exhibited significantly elevated peak areas (Supplementary Table 2): β-pinene showing the most pronounced increase (525.91%, *p* < 0.05), followed by limonene (121.15%, *p* < 0.01), 1-(1,5-dimethyl-4-hexenyl)-4-methyl-benzene (35.28%, *p* < 0.05), lavender lactone (35.71%, *p* < 0.05), 1,6-dimethyl-naphthalene (38.82%, *p* < 0.01), 1,8-dimethyl-naphthalene (13.78%, *p* < 0.05), and tetradecane (4.72%, *p* < 0.05). In contrast, the levels of (E,E)-3,5-octadien-2-one and butanoic acid were significantly decreased (Supplementary Table 2) by 16.05% (*p* < 0.05) and 38.71% (*p* < 0.01), respectively. It was demonstrated SA stress induces metabolic reprograming that favors the biosynthesis of terpenoid and aromatic compounds.

To better characterize VOCs changes across five wheat varieties cultivated in soils with different SA levels, the relative contents of key VOCs were measured, and the results are shown in Figure 1Bβ-pinene and limonene exhibited marked increases (Supplementary Table 3) by 474.09% (*p* < 0.05) and 109.74% (*p* < 0.01), respectively, confirming their roles as SA stress-responsive biomarkers. Conversely, tridecane, (E,E)-3,5-octadien-2-one and butanoic acid exhibited significant reductions (Supplementary Table 3): 10.17% (*p* < 0.05), and 42.95% (*p* < 0.01), respectively.

Based on the pairing results of peak areas and relative contents, β-pinene, limonene, (E,E)-3,5-octadien-2-one and butanoic acid could be regarded as important indicator compounds for wheat cultivation in SA soil. Given that wheat cultivated on SA soil has a relatively strong aroma, these compounds might also be the ones that contribute to the wheat aroma.

### 3.2 Molecular docking analysis

**FIGURE 2:**
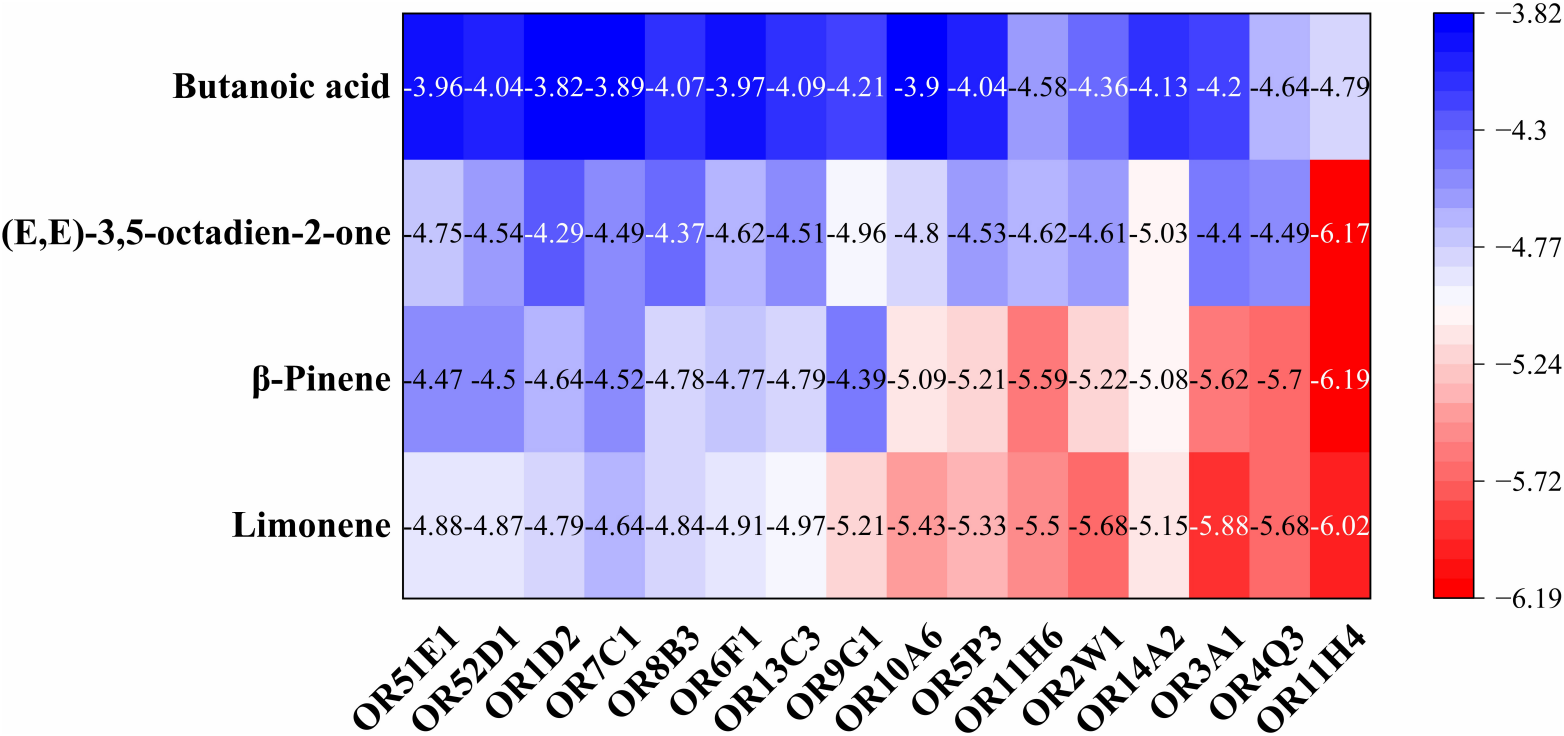
The binding energy heat map between the important compounds and the olfactory receptors.

To further investigate whether these four VOCs act as critical compounds for wheat cultivated in saline-alkali land, molecular docking was employed. In the docking simulations, a negative binding energy indicates spontaneous ligand-receptor binding, with its magnitude correlating directly to binding affinity. More negative values reflect stronger intermolecular interactions and higher probability of stable complex formation (25). Based on established criteria: binding energy < -4.25 kcal/mol suggests detectable binding activity, implying potential ligand-receptor recognition; binding energy < -5.0 kcal/mol signifies high binding affinity, likely corresponding to biologically relevant interactions (26). For this study, compounds meeting the threshold of binding energy < -4.25 kcal/mol were prioritized for further analysis. The binding energy heat map between the important compounds and the olfactory receptors (Figure 2) revealed that binding energy range of butanoic acid with all 16 olfactory receptors was -4.79 to -3.82 kcal/mol, and its affinity is relatively weak (> -5 kcal/mol), whereas limonene, β-pinene and (E,E)-3,5-octadien-2-one exhibited moderate to strong binding affinity, with binding energies of -6.02 to -4.64 kcal/mol, -6.19 to -4.39 kcal/mol, and -6.17 to -4.29 kcal/mol, respectively. These results align with prior evidence that such binding energies modulate receptor conformational dynamics and enhance agonist efficacy in flavor perception (27).

**FIGURE 3:**
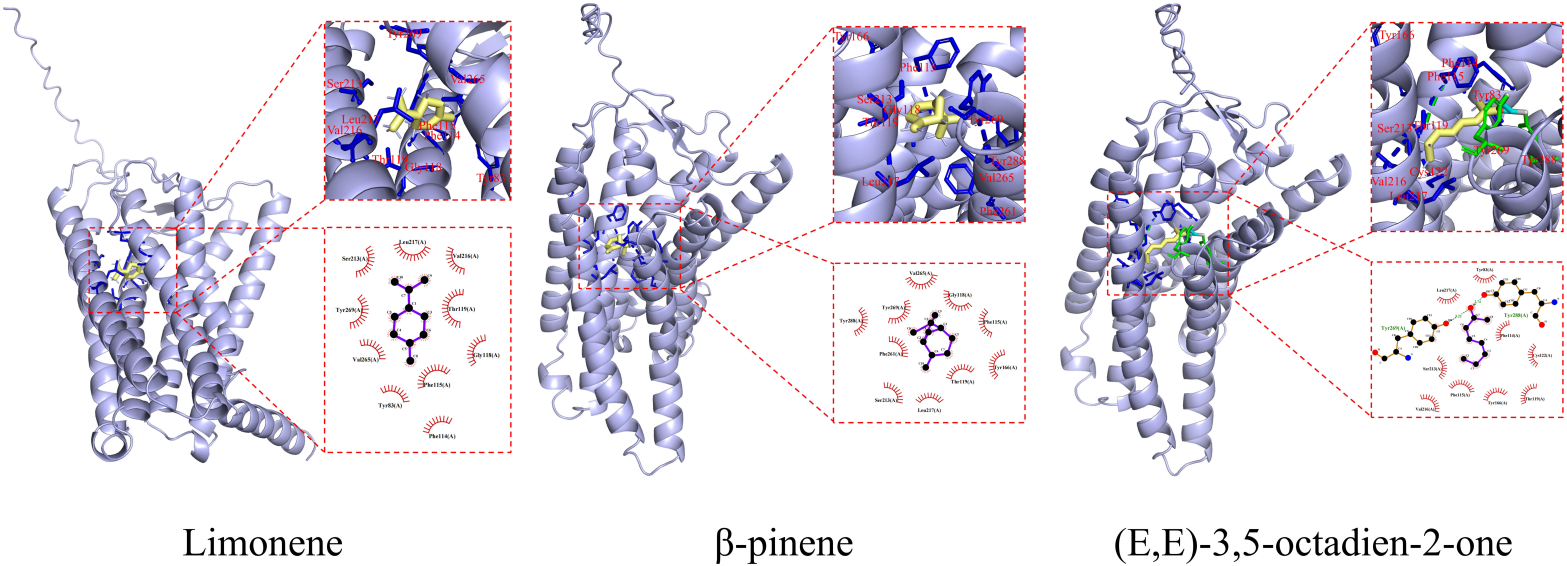
Binding sites and interactions for molecular docking between key volatile organic compounds (limonene, β-pinene, and (E,E)-3,5-octadien-2-one) and OR11H4.

Molecular docking studies have demonstrated that aroma compounds enhance food flavor by binding to potential active sites of ORs (28). Among the sixteen ORs examined, OR11H4 exhibited significantly stronger binding affinity (Figure 2), and was therefore selected to elucidate mutual binding interactions with three key compounds. As shown in Figure 3, fourteen interacting amino acid binding residues (Leu217, Ser213, Tyr269, Tyr83, Tyr166, Tyr288, Val265, Val216, Phe114, Phe115, Phe261, Gly118, Thr119, and Cys122) were identified in the OR11H4 binding porket. Notably, Leu217, Ser213, Tyr269, Phe115, and Thr119 served as common binding sites, suggesting their suggesting their critical role as key interaction residues (23). Furthermore, limonene formed hydrophobic interactions with Leu217, Ser213, Tyr269, Tyr83, Val265, Val216, Phe114, Phe115, Gly118, and Thr119; β-pinene bound via hydrophobic interactions to Leu217, Ser213, Tyr269, Tyr166, Tyr288, Val265, Phe115, Phe261, Gly118, and Thr119; while (E,E)-3,5-octadien-2-one established hydrogen bonds with Tyr269 and Tyr288, alongside hydrophobic interactions involving Leu217, Ser213, Tyr83, Tyr166, Val216, Phe114, Phe115, Thr119, and Cys122.

In summary, Leu217, Ser213, Tyr269, Phe115, and Thr119 are critical binding residues, with hydrophobic interactions and hydrogen bonds acting as primary driving forces in ligand-receptor recognition.

### 3.3 Volatile organic compounds analysis by relative odor activity values

The characteristic “wheat aroma” is influenced not only by the concentration of key odorants but also by their sensory threshold. Relative odor activity values (ROAVs) could be utilized for assessing the contribution of individual components toward the overall flavor (24). Based on established criteria (29), VOCs with ROAVs. > 1 dominate the overall flavor, while those with ROAVs between 0.1 and 1 exert modifying effects; VOCs with ROAVs. < 0.1 contribute negligibly. To identify key aroma compounds in the SA soil-cultivated wheat, ROAVs were calculated for limonene, β-pinene, and (E,E)-3,5-octadien-2-one. Consistently, ROAVs exceeded 1 across five wheat varieties grown in soils with distinct salinity-alkalinity gradients (Table 3), indicating their significant contribution to the characteristic flavor of SA-cultivated wheat.

Based on this premise, the three VOCs were considered as key flavor compounds that potentially contribute significantly to the overall flavor perception of “wheat aroma.” The proportion range of each component can be determined by calculating the percentage of peak area for each key flavor compound among all key flavor compounds, as presented in Supplementary Table 4.

### 3.4 Principal component analysis and linear discriminant analysis

**FIGURE 4:**
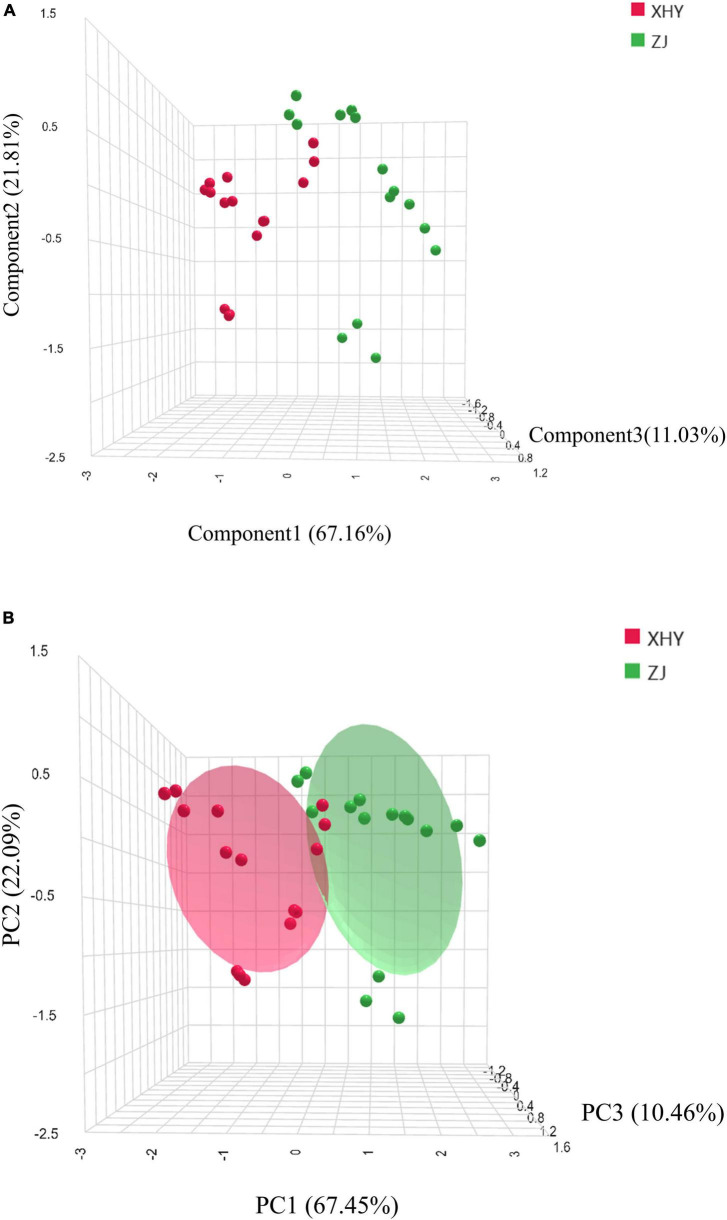
PCA **(A)** and LDA **(B)** of the selected 3 key volatile organic compounds in wheat cultivated in XHY and ZJ.

Principal component analysis (PCA), an unsupervised dimensionality reduction technique, extracts orthogonal components that maximize variance retention while approximating original variables. This method effectively identifies inter-group similarities and intra-group differences within complex datasets (30). PCA conducted on the three potential key aroma compounds revealed that the first three principal components (PC1-PC3) accounted for 67.45, 22.09, and 10.46% of total variance, respectively, with a cumulative variance contribution rate was 100% (Figure 4A). This confirms PC1-PC3 comprehensively capture the dominant variation patterns in the aroma profile. Loadings analysis further identified limonene, β-pinene and (E,E)-3,5-octadien-2-one could serve as reliable indicators for distinguishing XHY and ZJ cultivated wheat.

Complementarily, linear discriminant analysis (LDA)—a supervised technique optimizing inter-class separation while minimizing intra-class variance (31)—was employed to validate the classification efficacy of these three VOCs. Notably, LDA achieved 90.0% cross-validated accuracy in distinguishing wheat grown in distinct salinity-alkalinity soils. Projection plots (Figure 4B) demonstrated complete spatial segregation between XHY and ZJ samples along the first three discriminant functions (LD1-LD3), which collectively explained 100% of total variance. LD1 dominated class separation (67.16% variance), with limonene exhibiting the highest standardized coefficient (discriminant coefficient = 0.73), indicating its pronounced sensitivity to salinity gradients.

Synthesizing PCA and LDA outcomes, limonene, β-pinene, and (E,E)-3,5-octadien-2-one are established as signature flavor compounds defining the aroma signature of SA-cultivated wheat, and may also be the characteristic biomarkers of wheat aroma.

## 4 Discussion

The results show that limonene, β-pinene and (E,E)-3,5-octadien-2-one can serve as an reliable indicators for the identification of wheat cultivated in SA soil (Figure 1). This is in line with the more general notion that abiotic stress reshapes plant secondary metabolism (including volatile terpenoids and carbonyl compounds).

As shown in Table 3, β-pinene exhibited flavors of pine, resin, and turpentine, while limonene possessed sweet and orange notes (32). As monoterpenes, both compounds exhibit significant accumulation under SA stress (pH range: 8.14∼8.54; total salt content: 2.19∼2.89 g/kg) condition (Figure 1), aligning with established patterns of terpenoid enhancement in plants exposed to osmotic stressors. Plant monoterpenes were primarily synthesized through the 2-methyl-D-erythritol-4-phosphate pathway within plastids (33). Geranyl diphosphate served as a common precursor for all monoterpenes (34), which could be converted into β-pinene and limonene by various members of the terpene synthase/cyclase enzyme family (35). Although direct measurements of terpenoid volatile accumulation in wheat under saline-alkali stress are still limited, evidence suggest that wheat may cope with salt stress, at least in part, by modulating terpenoid biosynthetic pathways. Transcriptome analyses under salt stress have shown that pathways related to secondary metabolism, including terpenoid metabolism, are significantly enriched in the roots of salt-tolerant wheat genotypes (36). Similar stress-induced activation of terpenoid metabolic pathways has been reported in other plant species. According to report (14), an increased release of different terpenes in tomato plants with rising soil salt concentrations under salt stress conditions. It was found that there was an elevation in the content of certain terpenoids in salvia plants with increasing salt stress levels (37). Other studies also demonstrated that moderate drought could enhance plant terpenoids content (15). Stress treatment was carried out on Jinxuan tea tree varieties, and it was found that upregulation in gene expression related to the metabolic pathway involved in terpene carbon skeleton synthesis. This led to enhanced accumulation of precursors for terpenoid biosynthesis and increased volatile compound content (38). These findings may explain the elevated levels of these two monoterpenes under SA stress conditions.

Conversely, (E,E)-3,5-octadien-2-one content decreased markedly in SA-cultivated wheat, mirroring the suppression of 1-octen-3-one reported saline–alkali soil (19). This reduction likely stems from salt-triggered lipoxygenase (LOX) activation (39), which accelerates peroxidation of unsaturated fatty acids (e.g., linoleic/linolenic acid) (40) to generate hydroperoxide intermediates. These intermediates are preferentially metabolized to C6 aldehydes and alcohols (e.g., hexanal, 1-octen-3-ol) via hydroperoxide lyases (HPLs) and alcohol dehydrogenases (ADHs) (41), rather than to unsaturated ketones like (E,E)-3,5-octadien-2-one. Additionally, enzymatic reduction of (E,E)-3,5-octadien-2-one to its less volatile alcohol derivative may further deplete its concentration under oxidative stress. This metabolic rerouting reflects a broader reconfiguration of oxylipin networks under salinity, prioritizing compounds with lower flavor thresholds or stress-protective roles.

This study primarily focuses on the characteristics of VOCs in wheat kernels, while acknowledging that the VOCs of derived products, such as flour and cooked wheat-based foods, also warrant investigation. Future studies should (i) validate these biomarkers over many years and in multiple varieties to confirm their stability; (ii) analyze the effects of different saline-alkali stress environments and the spatial position of wheat grains on the characteristic VOCs of wheat aroma; and (ii) Conduct sensory-guided gas chromatography-olfactometry (GC-O) and recombination/omission experiments to directly confirm the contribution of the identified biomarkers to wheat aroma. These steps will help to further validate and refine the proposed biomarkers in future work.

## 5 Conclusion

In this study, the VOCs of five wheat varieties cultivated in soils with varying levels of salinity and alkalinity were analyzed by HS-SPME-GC-MS. Pairwise quantification revealed significant alterations (*p* < 0.05) in β-pinene, limonene, (E,E)-3,5-octadien-2-one, and butanoic acid, identifying them as diagnostic biomarkers for SA-cultivated wheat. Molecular docking further established β-pinene, limonene, and (E,E)-3,5-octadien-2-one as flavor determinants by exhibiting high-affinity binding (< -4.25 kcal/mol) to olfactory receptors, driven by hydrophobic interactions with residues Leu217, Ser213,Phe115, and Thr119 and hydrogen-bond networks involving Tyr269. Synergistically, ROAV analysis (> 1) identified these compounds as key biomarkers of “Wheat Aroma” in saline-alkali (SA) soil-cultivated wheat. These biomarkers serve as potential indicators, contributing to the understanding of the metabolic reprograming patterns in wheat under salinity-alkalinity stress.

## Data Availability

The original contributions presented in the study are included in the article/Supplementary material, further inquiries can be directed to the corresponding authors.
