# Supplementary material for: Wheat aroma biomarkers induced by saline-alkali soil: based on HS-SPME-GC-MS and molecular docking
Source: Front Nutr. 2026 Mar 4;13:1788319. doi: 10.3389/fnut.2026.1788319 (PMC12996116; doi:10.3389/fnut.2026.1788319)
Supplement: Supplementary file 1 [file Data_Sheet_1.DOCX]

**Table S1 VOCs with significant differences in peak area between XHY and ZJ（×10^6^）**

| **Volatile organic compounds** | **C6002** | | **C6005** | | **JM22** | | **N01** | | **SL02-1** | |
| --- | --- | --- | --- | --- | --- | --- | --- | --- | --- | --- |
|  | **XHY** | **ZJ** | **XHY** | **ZJ** | **XHY** | **ZJ** | **XHY** | **ZJ** | **XHY** | **ZJ** |
| beta-pinene | 4.768±0.291 | 20.405±3.927  ** | 0.600±0.097 | 13.659±1.831  ** | 2.977±0.403 | 12.746±1.864  ** | - | 1.752±0.187  ** | 0.187±0.070 | 4.840±0.434  ** |
| Limonene | 94.908±9.525 | 124.815±1.247  ** | 53.205±4.297 | 122.049±2.13  ** | 3.071±0.285 | 50.74±5.012  ** | 39.02±2.796 | 105.833±5.782  ** | 48.367±5.26 | 124.167±3.115  ** |
| Tetradecane | 50.852±2.062 | 51.424±2.824 | 45.911±0.642 | 46.530±0.580 | 48.317±2.955 | 51.920±6.343 | 53.991±2.492 | 56.640±2.942 | 54.907±3.042 | 59.446±1.793 |
| (E,E)-3,5-octadien-2-one | 7.074±0.118  ** | 6.233±0.063 | 6.572±0.033 | 6.51±0.073 | 7.249±0.091  ** | 5.41±0.411 | 9.165±0.173  ** | 7.411±0.26 | 8.175±0.34  ** | 6.533±0.171 |
| butanoic acid | 0.555±0.027  ** | 0.381±0.011 | 0.522±0.015  ** | 0.389±0.011 | 0.324±0.018  ** | 0.233±0.02 | 0.436±0.025  ** | 0.238±0.016 | 0.411±0.022  ** | 0.138±0.01 |
| lavender lactone | 0.803±0.017 | 0.930±0.030  ** | 0.906±0.016 | 0.959±0.021  * | 0.695±0.006 | 1.174±0.031  ** | 0.551±0.012 | 0.745±0.008  ** | 0.590±0.025 | 1.003±0.028  ** |
| Benzene,1-(1,5-dimethyl-4-hexenyl)-4-methyl- | 0.201±0.088 | 0.225±0.035 | 0.133±0.013 | 0.134±0.022 | 0.160±0.008 | 0.171±0.083 | 0.085±0.008 | 0.117±0.016  ** | 0.090±0.008 | 0.112±0.014 |
| 1,8-dimethyl-naphthalene | 0.783±0.060 | 1.254±0.046  ** | 0.726±0.044 | 1.113±0.062  ** | 0.626±0.066 | 0.765±0.039  * | 0.792±0.015 | 1.058±0.094  * | 1.107±0.026 | 1.264±0.106 |
| 1,6-dimethyl-naphthalene | 0.259±0.018 | 0.431±0.012  ** | 0.240±0.012 | 0.379±0.024  ** | 0.211±0.02 | 0.264±0.012  * | 0.276±0.01 | 0.371±0.034  * | 0.374±0.013 | 0.442±0.04  * |

Notes: Data in this table are showed by means ± standard deviations; * and ** represent the significant difference (p＜0.05) and (p＜0.01) respectively; "-" indicates that the system is not detected..

**Table S2 VOCs with significant differences in relative content of peak area between XHY and ZJ**

| **Volatile organic compounds** | **C6002** | | **C6005** | | **JM22** | | **N01** | | **SL02-1** | |
| --- | --- | --- | --- | --- | --- | --- | --- | --- | --- | --- |
|  | **XHY** | **ZJ** | **XHY** | **ZJ** | **XHY** | **ZJ** | **XHY** | **ZJ** | **XHY** | **ZJ** |
| beta-pinene | 0.543±0.037 | 2.304±0.468  ** | 0.076±0.011 | 1.619±0.221  ** | 0.4±0.044 | 1.337±0.214  ** | - | 0.192±0.022  ** | 0.021±0.008 | 0.52±0.041  ** |
| Limonene | 10.805±1.096 | 14.08±0.339  ** | 6.772±0.467 | 14.459±0.189  ** | 0.414±0.049 | 5.319±0.549  ** | 4.508±0.3 | 11.569±0.467  ** | 5.526±0.528 | 13.355±0.311  ** |
| Tridecane | 4.991±0.139  * | 4.695±0.08 | 5.29±0.104  * | 4.344±0.134 | 5.389±0.189 | 5.273±0.248 | 5.094±0.09  * | 4.794±0.116 | 5.975±0.091  ** | 4.913±0.157 |
| (E,E)-3,5-octadien-2-one | 0.805±0.014  ** | 0.703±0.017 | 0.837±0.01  ** | 0.771±0.018 | 0.975±0.024  ** | 0.566±0.005 | 1.059±0.013  ** | 0.81±0.022 | 0.934±0.009  ** | 0.703±0.015 |
| butanoic acid | 0.063±0.003  ** | 0.043±0 | 0.067±0.003  ** | 0.046±0.002 | 0.044±0.001  * | 0.025±0.004 | 0.05±0.002  * | 0.026±0.001 | 0.047±0.001  * | 0.015±0.001 |

Notes: Data in this table are showed by means ± standard deviations; * and ** represent the significant difference (p＜0.05) and (p＜0.01) respectively; "-" indicates that the system is not detected.

**Table S3 Peak area percentage of key VOCs of wheat cultivated in XHY and ZJ**

| **Volatile organic compounds** | **C6002** | | **C6005** | | **JM22** | | **N01** | | **SL02-1** | |
| --- | --- | --- | --- | --- | --- | --- | --- | --- | --- | --- |
|  | **XHY** | **ZJ** | **XHY** | **ZJ** | **XHY** | **ZJ** | **XHY** | **ZJ** | **XHY** | **ZJ** |
| β-pinene | 4.49±0.39 | 13.44±2.29  ** | 0.99±0.11 | 9.6±1.29  ** | 22.37±2.8 | 18.44±1.05 | 0±0 | 1.53±0.21  ** | 0.32±0.09 | 3.57±0.23  ** |
| limonene | 88.86±0.85  ** | 82.45±2.19 | 88.09±0.76 | 85.82±1.32 | 23.11±2.36 | 73.66±0.16  ** | 80.93±1.23 | 92.02±0.49  ** | 85.2±1.04 | 91.61±0.12  ** |
| (E,E)-3,5-octadien-2-one | 6.66±0.52  ** | 4.12±0.11 | 10.92±0.82  ** | 4.58±0.08 | 54.52±0.74  ** | 7.9±0.91 | 19.07±1.23  ** | 6.45±0.28 | 14.48±1.14  ** | 4.82±0.19 |

Note: Data in this table are showed by means ± standard deviations; * and ** represent the significant difference (*p*＜0.05) and (*p*＜0.01) respectively.


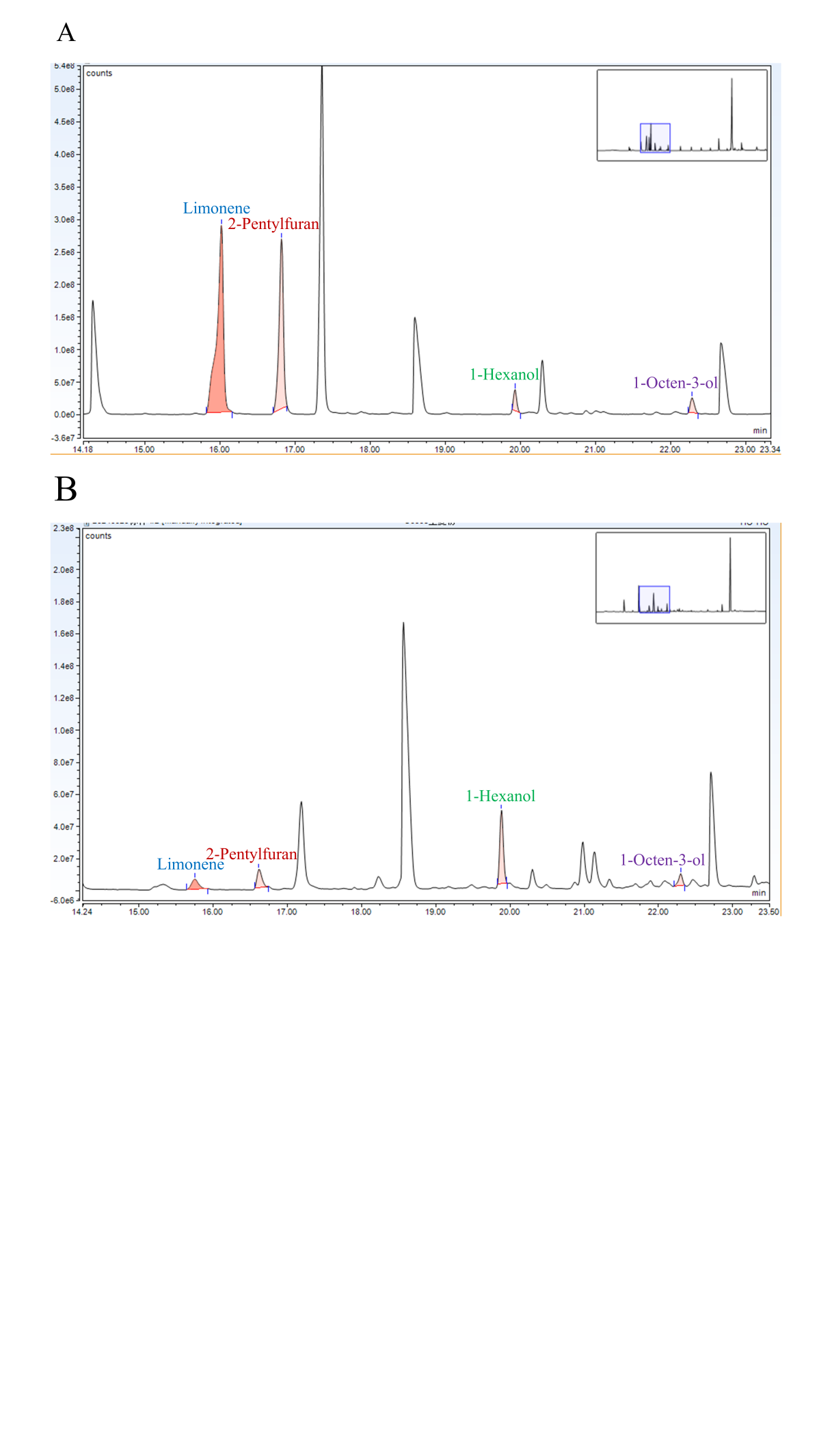


**Figure S1.** Partial ion current chromatograms were obtained for the volatile organic compounds of the mix standard sample (A) and test sample (C6005 as an example) (B).
